# Design of a UWB Interference-Rejection LNA Based on a Q-Enhanced Notch Filter

**DOI:** 10.3390/mi16121389

**Published:** 2025-12-07

**Authors:** Jiaxuan Li, Yuxin Fan, Fan Meng

**Affiliations:** Nanjing Research Institute of Electronics Technology, Nanjing 210039, China; lijiaxuan2956@163.com (J.L.);

**Keywords:** active capacitance, GaAs, interference rejection, low-noise amplifier, notch filter, UWB

## Abstract

A Q-enhanced notch filter for interference-rejection LNAs is proposed in this brief. The active capacitance is introduced into the notch filter to improve the quality factor by the negative resistance effect. The designed notch filter achieves excellent performance with a narrow attenuation bandwidth from 5.75 GHz to 5.95 GHz, which can be applied to suppress interference from the IEEE 802.11a. To validate the feasibility of the proposed trap filter in both GaAs process technology and principle, a 3–15 GHz ultra-wideband low-noise amplifier was designed and fabricated using a 0.15-micron gallium arsenide pseudomorphs field-effect transistor process. The frequency-dependent feedback loops are employed between gate and drain stages for wideband input matching and gain flatness. The notch filter is inserted between two stages of the LNA. The measurement results show that the interference-rejection LNA achieves a maximum gain of 24.5 dB and a minimum noise figure of 1.8 dB in the operating band. The notch filter has a maximum interference-rejection ratio of 35.2 dB at 5.8 GHz with almost no effect on the desired gain of the LNA. The LNA has a power consumption of 168 mW, including the notch filter with a size of 1.93 × 0.72 mm^2^.

## 1. Introduction

UWB systems, which are announced by the Federal Communications Commission in the 3.1–10.6 GHz [1], have been widely used in modern wireless communications due to low power level, high data-rate transmission (up to 480 Mb/s), and UWB standards for short-range connectivity [2,3]. However, the power spectral density of UWB signals is limited to −41.3 dBm/MHz, which makes it particularly susceptible to interference from some high-power narrowband systems in the band [4,5]. For instance, ranging from 5.7 GHz to 5.85 GHz, the interference sources from the wireless local area network (WLAN) IEEE 802.11a [6] are more than 60 dB higher than the received UWB signal power, resulting in desensitization of the received link [7]. Therefore, to prevent received UWB signals from such detrimental effects, the frequencies of narrowband systems need to be avoided [8].

The interference rejection can be effectively achieved by inserting an off-chip notch filter in front of the LNA, but it will occupy an additional area. Although the integration of notch filters in LNAs can overcome this problem, the low-quality factors (*Q_s_*) of on-chip spiral inductors are the major obstacle to achieving high-performance notch filters [9].

There have been many studies reported on the interference-rejection LNA (IR-LNA) in the past decade [10,11,12,13,14,15,16,17]. In [10,11,12], passive LC notch filters are introduced into the LNAs to reject out-of-band interference. However, these filters are inappropriate for UWB LNAs since the lower *Q_s_* of notch filters will worsen the in-band gain. A tunable on-chip synthesized notch filter is designed in [13] based on a balanced amplifier idea. The filter has a sharp notch and an excellent interference-rejection ratio (IRR). Moreover, utilizing the negative resistance effect of transistors, the LNA integrated with a high *Q* notch filter is realized in [14], which achieves the in-band interference rejection at 5.2 GHz with a slight deterioration in the desired gain. In [15], the passive inductor in the conventional dual resonant notch filter is replaced by active circuits; simultaneously achieving the out-of-band interference rejection of LNA at both high and low frequencies is achieved. The active third-order notch filter is designed in [16], which provides an additional pole to compensate for the desired band gain. In [17], the feedback technique is applied to the active notch filter to enhance the *Q* and decrease the power consumption. These active notch filters both utilize the negative resistance effect of the transistors to enhance the *Q*, thus avoiding deterioration of the desired gain.

**Figure 1:**
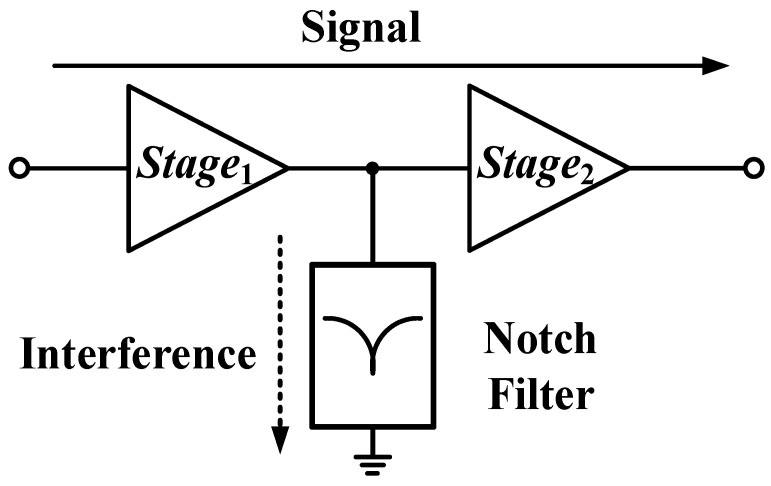
The topology diagram of the IR-LNA with Q-enhanced notch filters.

In this brief, an IR-LNA integrated with an on-chip notch filter is designed and fabricated in a 0.15-μm GaAs process, as shown in Figure 1. Thereinto, the active capacitance is introduced into the notch filter to enhance the *Q* through the negative resistance effect. The designed active notch filter exhibits an IRR of 35.2 dB at 5.8 GHz with little deterioration of the desired gain. This excellent performance makes this notch filter suitable for IR-LNA designs.

## 2. Design of the Q-Enhanced Notch Filter

**Figure 2:**
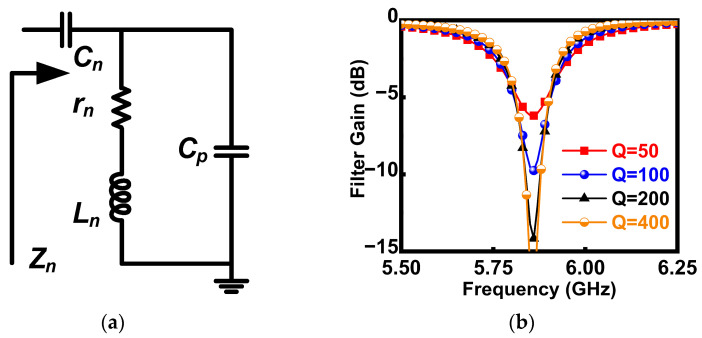
(**a**) The schematic of the third-order LC resonator. (**b**) The simulated results of the third-order notch filter at different *Q* of *L_n_*.

The notch filter is designed with reference to the third-order LC passive resonator in [18], as shown in Figure 2. When ignoring the passive loss *r_n_* of *L_n_*, the input impedance of the resonator is given by

(1) $$Z_{n}=\frac{1+s^{2}\left(C_{n}+C_{p}\right)L_{n}}{sC_{n}\left(1+s^{2}C_{p}L_{n}\right)}$$

*L_n_*, *C_n_*, and *C_p_* constitute the components of a third-order LC passive notch filter, while *R_n_* represents the parasitic resistance of the inductor *L_n_*. From (1), a pole *f_p_* and a zero *f_n_* can be, respectively, obtained as

$$f_{p}=\frac{1}{2\pi \sqrt{L_{n}C_{p}}}\begin{array}{cc} & f_{n}=\frac{1}{2\pi \sqrt{L_{n}\left(C_{p}+C_{n}\right)}} \end{array}$$

*Z_n_* shows low impedance at *f_p_* to realize the interference rejection. In addition, the parallel resonance introduced by *C_p_* makes *Z_n_* present high impedance at *f_p_*, which contributes to avoiding the deterioration of the desired gain. The notch depth is closely dependent on the *Q_s_* (*Q* = *ωL_n_*/*r_n_*) of passive components, on-chip spiral inductors especially [17]. Figure 2b shows the simulated results of notch performance at different *Q* of *L_n_*, which indicates that the *L_n_* with low *Q* will make the notch filter ineffective. However, the *Q* factor of inductors used in GaAs processes ranges between 20 and 40, which complicates the design of on-chip notch filters. Therefore, it is necessary to compensate for the loss of passive inductors when designing on-chip notch filters, and this can be achieved by utilizing the negative resistance effect of transistors [14,15,16,17].

**Figure 3:**
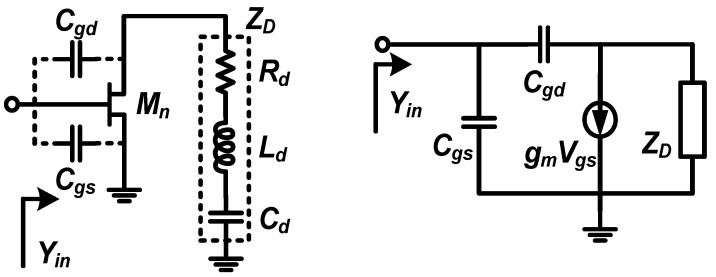
The schematic of the active capacitance and its equivalent circuit.

The active capacitance referenced in [19], which consists of a transistor and a series RLC circuit, is designed to provide the necessary negative resistance of the notch filter. The schematic of it is shown in Figure 3. In this circuit, the input admittance can be derived as follows:

(2) $$Y_{in}=\underset{I}{\underbrace{\frac{R_{d}+g_{m}\left(R_{d}^{2}+\left(X_{d}-X_{2}\right)X_{d}\right)}{\zeta }}}+j\underset{\mathrm{II}}{\underbrace{\left(\frac{1}{X_{1}}-\frac{X_{d}-X_{2}+g_{m}X_{2}R_{d}}{\zeta }\right)}}$$

where the parameters are

$$\begin{array}{l} \begin{array}{cc} X_{1}=1/\omega C_{gs} & X_{2}=1/\omega C_{gd} \end{array}\\ X_{d}=\omega L_{d}-1/\omega C_{d}\\ \zeta =R_{d}^{2}+{\left(X_{d}-X_{2}\right)}^{2} \end{array}$$

From (2), the precondition of negative resistance can be obtained by setting I < 0, which can be derived as

(3) $$\begin{array}{c} 0<\frac{X_{2}-\sqrt{\Delta_{d}}}{2}<X_{d}<\frac{X_{2}+\sqrt{\Delta_{d}}}{2}<X_{2}\\ \begin{array}{c} \Delta_{d}=X_{2}^{2}-\frac{4\left(1+g_{m}R_{d}\right)}{g_{m}}R_{d} \end{array} \end{array}$$

To ensure Δ*_d_* > 0, *R_d_* is limited to

(4) $$R_{d}<\frac{\sqrt{1+g_{m}^{2}X_{2}^{2}}-1}{2g_{m}}$$

**Figure 4:**
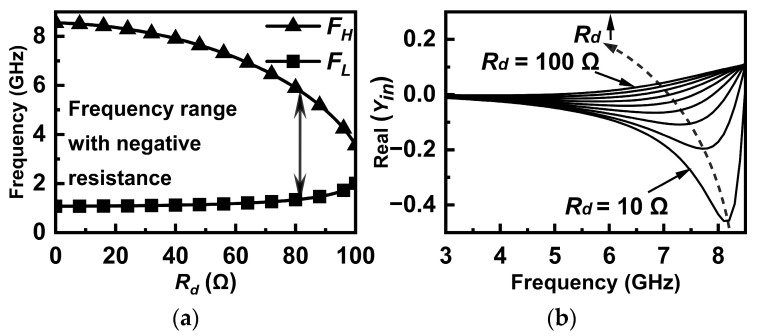
The simulated results of (**a**) negative resistance bandwidth and (**b**) values at different *R_d_*.

**Figure 5:**
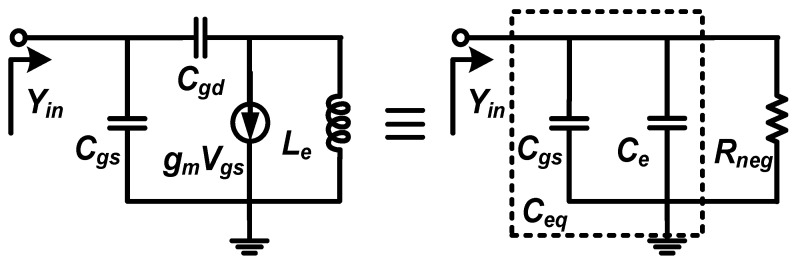
The equivalent circuit of the active capacitance with the conditions of (3) and *R_d_* = 0.

According to (4), the maximum value of *R_d_* is dominated by the size and bias of *M_n_*. Figure 4a and Figure 4b, respectively, shows the simulated results of the negative resistance bandwidth and values at different *R_d_*. It can be seen that a larger *R_d_* not only makes the negative resistance bandwidth narrow but also results in the degeneration of the negative resistance effect. Moreover, II > 0 is unconditionally satisfied in the case of (3), which means the active circuit in Figure 3 can be equivalent to a parallel connection of a capacitor *C_eq_* and a negative resistor *R_neg_*. To simplify the analysis, assume *Z_D_* = *jωL_e_* with the condition that *R_d_* = 0, where *L_e_* is the equivalent inductor value of *Z_D_* under the condition of (3). The circuit of the active capacitance is shown in Figure 5, where the parameters are

(5) $$\begin{array}{cc} C_{e}=\frac{C_{gd}}{1-\omega^{2}L_{e}C_{gd}}, & R_{neg}= \end{array}\frac{\omega^{2}L_{e}C_{gd}-1}{\omega^{2}g_{m}L_{e}C_{gd}}$$

From (5), both increasing *C_gd_* and *C_gs_* make *C_eq_* larger and *R_neg_* less with the precondition that *ω*^2^*L_e_C_gd_* < 1. Therefore, we can obtain an initial frequency range of the negative resistance through *L_d_* and *C_d_*. Then the negative resistance bandwidth can be controlled by *R_d_*. The values of negative resistance and reactance based on *L_d_*, *C_d_*, and *R_d_* can be further tuned by setting different biases or sizes of *M_n_*.

**Figure 6:**
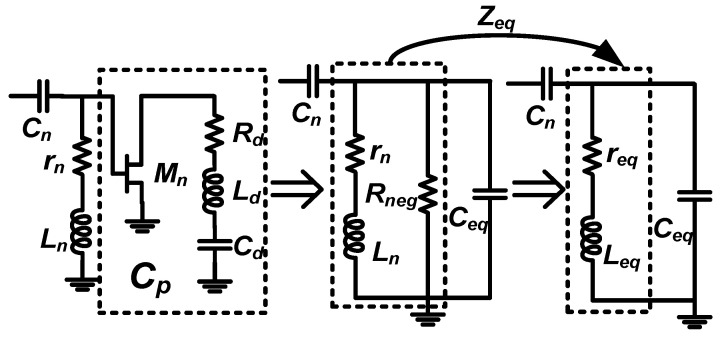
The schematic of a notch filter with active capacitance.

The *C_p_* in Figure 2a is replaced with the designed active capacitance, as shown in Figure 6. The *Z_eq_* can be expressed as (6). From (6), we can find that the *r_eq_* = 0 is satisfied under the condition of (7). Therefore, the active capacitance has the potential to improve the *Q* of the notch filter.

(6) $$\begin{array}{c} Z_{eq}=\underset{r_{eq}}{\underbrace{\frac{R_{neg}\left(r_{n}^{2}+r_{n}R_{neg}+\omega^{2}L_{n}^{2}\right)}{\sigma }}}+j\omega \underset{L_{eq}}{\underbrace{\frac{L_{n}R_{neg}^{2}}{\sigma }}}\\ \sigma ={\left(r_{n}+R_{neg}\right)}^{2}+\omega^{2}L_{n}^{2} \end{array}$$

(7) $$R_{neg}=-(r_{n}+\frac{\omega^{2}L_{n}^{2}}{r_{n}})$$

**Figure 7:**
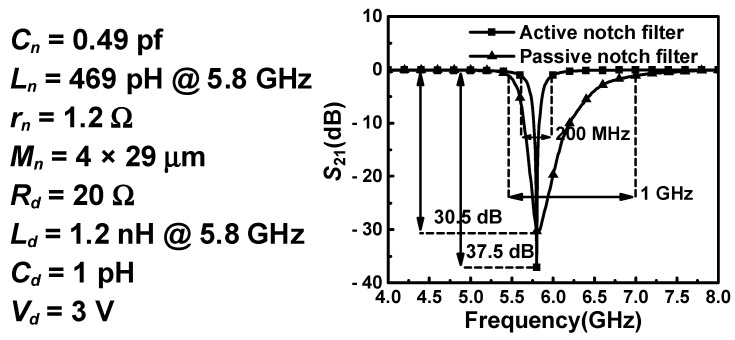
Comparison of S21 simulation results for active and passive notch filters and some key parameters of the active notch filter.

To verify the above theory, the active notch filter shown in Figure 6 is simulated, and its results are demonstrated in Figure 7. Compared with the passive notch filter, the performance of the active notch filter is significantly improved, which indicates that the active capacitance is helpful to enhance the *Q* factor of the notch filter.

## 3. Design of the Interference-Rejection Broadband LNA

**Figure 8:**
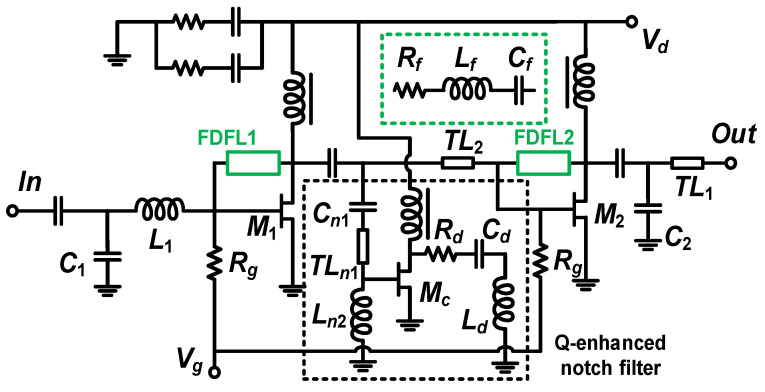
The schematic of the 3–15 GHz interference-rejection LNA with a Q-enhanced notch filter and frequency-dependent feedback loop (FDFL).

For verifying the feasibility of the designed Q-enhanced notch filter, we designed an LNA for UWB systems using the PL15-12 InGaAs pHEMT(WIN, Taiwan, China) process, as shown in Figure 8. The broadband LNAs of UWB systems are larger than three octaves, and it is difficult to realize such a wide bandwidth with a conventional common-source structure, so it is necessary to introduce some bandwidth-enhancing techniques to realize a broadband design.

The gain roll-off of transistors is one of the most important limiting factors in the broadband design. There are usually two ways to improve transistor gain roll-off: one is to reduce the gain in the low-frequency band through a feedback network to achieve gain flattening over a wide bandwidth range, and the other is to compensate for the high-frequency gain to achieve broadband gain flattening. One of the simplest and most effective methods is to adjust the broadband gain by introducing a negative feedback network [20]. Therefore, a frequency-dependent feedback loop (FDFL) is introduced into the design to achieve gain flattening over a wide bandwidth. In addition, the FDFL network can be equated to a lossy matching network, which helps to realize broadband impedance matching. At the same time, the stable operation of the circuit can be guaranteed due to the introduction of the lossy network.

**Figure 9:**
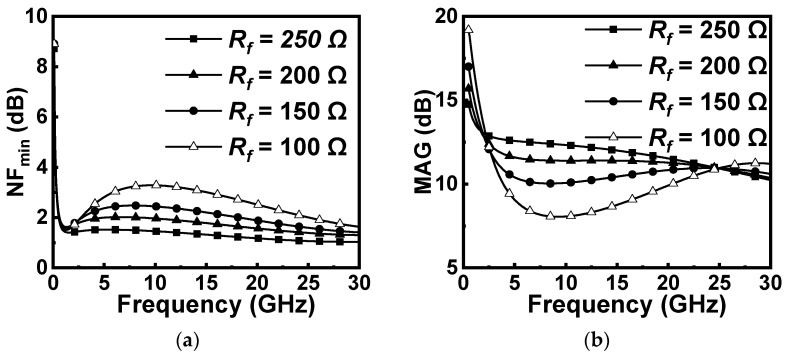
The effect of different Rf values on (**a**) NF and (**b**) MAG in the FDFL.

For cascaded LNA design, both the first and second stages are designed to achieve low noise with acceptable power gain. To take a proper value of the *R_f_*, we simulated the effect of different *R_f_* values on maximum available gain (MAG) and noise figure (NF), as shown in Figure 9. In this design, the introduction of FDFL inevitably leads to an increase in NF. To obtain a small NF for the designed broadband LNA, it is necessary to maximize the value of the feedback resistor in the first-stage FDFL network while ensuring stable operation of the amplifier [20]. Therefore, *R_f_* in the first stage of the FDFL is selected to be 340 Ω. Also, when the *R_f_* in the first FDFL is selected, the flatness of the overall gain can be adjusted by adjusting the *R_f_* in the second FDFL. Finally, an overall optimization is carried out for optimum circuit performance. Table 1 lists some of the optimized parameters of the key components.

## 4. Measurement Results

**Figure 10:**
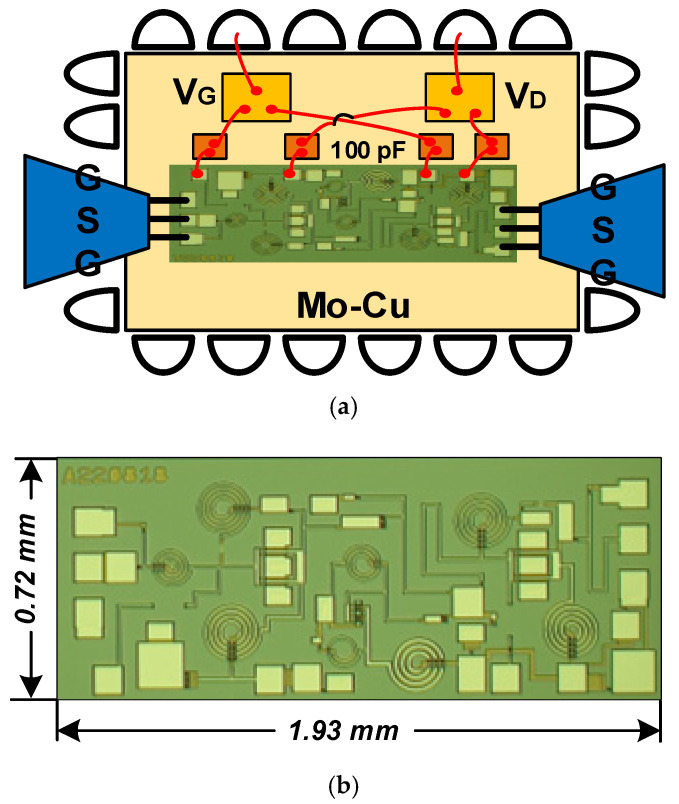
The proposed LNA MMIC: (**a**) schematic of the chip in QFN package for testing and (**b**) micrograph of the chip.

This design was fabricated using WIN’s 0.15 µm GaAs pHEMT process(WIN, Taiwan, China). A schematic of the QFN-packaged chip used for testing is shown in Figure 10a, and a micrograph of the chip is shown in Figure 10b, with an area of 1.93 × 0.72 mm^2^ (including all input/output test pads). The measurement setup includes a probe station (Cascade Summit 11000M), a vector network analyzer (Agilent N5244A), and a noise analyzer (Keysight N8976B). The LNA is biased at V_D_ = 3 V, V_G_ = −0.6 V for a total current of 56 mA.

**Figure 11:**
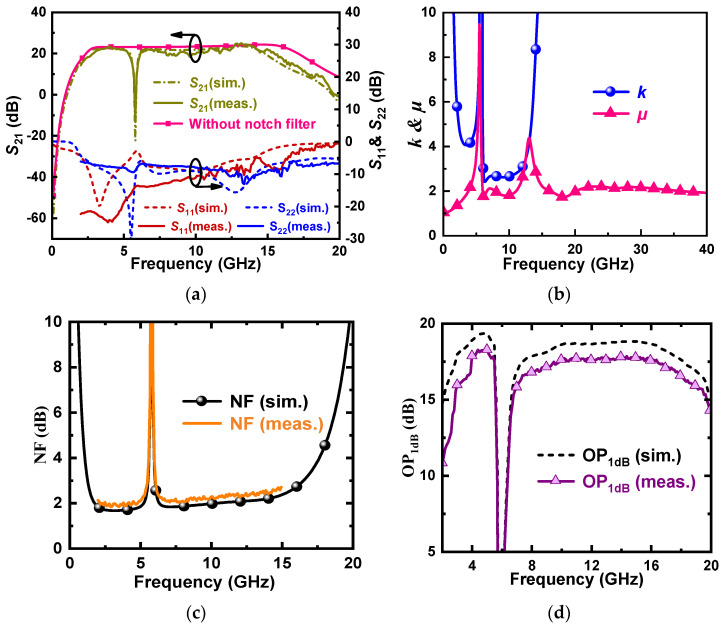
Measured and simulated (**a**) S-parameters. (**b**) Rollet k factor and stability factor *μ*. (**c**) NF. (**d**) OP1dB.

Figure 11a shows the S-parameters of the designed LNA. Compared with the simulation results, the measured S11 and S22 deteriorate slightly but are still less than 6 dB in the range of 3–15 GHz. S21 is about 1 dB lower than the simulated value, with an average value of 20.91 dB. In addition, S21 of the LNA without the Q-enhanced notch filter is given to visualize the interference suppression effect. The comparison shows that the maximum IRR of the designed LNA is 35.2 dB at 5.8 GHz, with 10 dB deterioration compared with the simulation results. However, the actual test results show that the introduction of the Q-enhanced notch filter has less effect on the in-band gain, and the trap frequency is shifted by only 0.05 GHz to the lower frequencies.

Figure 11b shows the simulated stability curves; the coefficients k and μ are both greater than 1 over the passband, implying that the LNA is unconditionally stable.

Figure 11c shows the simulated and the measured NF. The comparison shows that the NF of the measured deteriorates slightly compared to the simulated result, but the overall NF is lower than 2.6 dB, with a minimum value of 1.8 dB. It can also be derived that the introduction of the Q-enhanced notch filter does not have a significant impact on the in-band NF and only deteriorates the noise in a very narrow bandwidth of around 5.8 GHz, which is exactly what we expect.

Figure 11d shows the output 1 dB compression point (OP1dB), which has an average value of 11.82 dBm. Compared with the simulation results, the trend of the OP1dB curve in the 3–15 GHz range is in good agreement, but the overall OP1dB is reduced by about 2 dB.

These measurements validate the feasibility of the Q-enhanced notch filter. Table 2 summarizes the performance of the proposed LNA. Compared with other wideband interference-rejection LNAs, this design has a higher gain and lower noise figure. In addition, the Q-enhanced notch filter of this design minimizes the impact on the in-band gain compared to similar products.

## 5. Conclusions

In this brief, we present a 3–15 GHz interference-rejection low-noise amplifier using a 0.15 µm GaAs pHEMT process. The Q-enhanced notch filter with active capacitance is introduced in the LNA, which realizes a maximum interferer-rejected ratio of 35.2 dB at 5.8 GHz with small deteriorations for in-band gains. In addition, the LNA achieves a maximum gain of 24.5 dB and a minimum noise figure of 1.8 dB in the operating frequency range from 3 to 15 GHz. This demonstrates the suitability of the designed Q-enhanced notch filter with active capacitance for the design of an interference-rejection LNA.

## Figures and Tables

**Table 1:** Key Component Parameters of the Q-Enhanced LNA.

Parameters	*C*_1_	*L*_1_	*R_f_*_1_	*L_f_*_1_	*C_f_*_1_
Values	47 fF	824 pH	340 Ω	2.11 nH	1.89 pF
Parameters	*C_n_*_1_	*TL_n_*_1_	*L_n_*_2_	*M_c_*	*R_d_*
Values	0.11 pF	130 μm	2.74 nH	4 × 29 μm	62 Ω
Parameters	*C_d_*	*L_d_*	*R_f2_*	*L_f_*_2_	*C_f_*_2_
Values	0.33 fF	1.41 nH	237 Ω	2.81 nH	1.73 pF
Parameters	*M*_1_	*M*_2_	*V_D_*	*V_G_*	*C*_2_
Values	4 × 75 μm	4 × 100 μm	3 V	−0.6 V	46.8 fF

**Table 2:** Comparison with Other Interference-Rejection Broadband Low Noise Amplifiers.

Ref.	Technology	Filter Type	Frequency (GHz)	Gain * (dB)	NF (dB)	OP1dB (dBm)	Suppression ^#^ (dB)	P_dc_ (mW)	Area (mm^2^)
[14]	0.18 μm CMOS	Tunable active notch filter	3.1–10.6	13.2	6.2	−11	8.2@ 5.2 GHz	12.8	1.41
[15]	0.18 μm CMOS	Dual-band notch filter	2.8–6.2	11.5	3.8	6	25@ 1.8 GHz 32@ 8.5 GHz	2.5	0.62
[16]	0.18 μm CMOS	Third-order active notch filter	1.2–9.5	14.7	5.3	---	35.7@ 5.8 GHz	16	0.51
[17]	0.13 μm CMOS	Feedback notch-enhanced technique	3.3–10.1	16.05	3.7	---	40.9@ 5.77 GHz	10.2	0.88
[21]	0.13 μm CMOS	Inter-stage parallel resonator	14–16	10.8	4.2	---	38.5@ 24.3 GHz	5.2	---
This work	0.15 μm GaAs	Q-enhanced active notch filter	3–15	24.5	2.6	11.82	35.2@ 5.8 GHz	168	1.39

* Peak gain; **^#^** Suppression = |*S*_21_|_max_[dB] − |*S*_21_|_@*f* GHz_[dB].

## Data Availability

The original contributions presented in this study are included in the article. Further inquiries can be directed to the corresponding author.
